# Mission Roshni: Lighting up the world of India's children

**Published:** 2017

**Authors:** Shubhrakanti Bhattacharya, Sabitra Kundu, Prem Kumar SG, Elizabeth Kurian

**Affiliations:** Senior Manager, Program Development, Mission of Vision, Mumbai, India; Head, Program Development, Mission for Vision, Mumbai, India; Manager, Research, Mission for Vision, Mumbai, India; Chief Executive Officer, Mission for Vision, Mumbai, India

**Figure:**
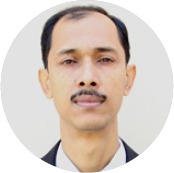
Shubhrakanti Bhattacharya

**Figure:**
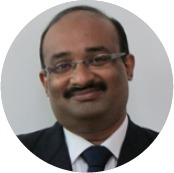
Sabitra Kundu

**Figure:**
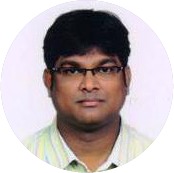
Prem Kumar SG

**Figure:**
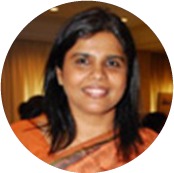
Elizabeth Kurian

**Childhood blindness is a priority because of the number of years of blindness. It is estimated to be the second leading cause of years of blindness after cataract.**

**Figure:**
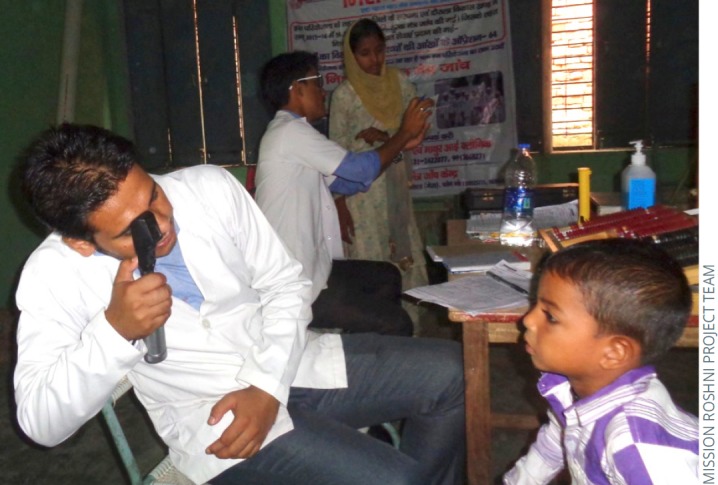
A boy being screened as part of this study. INDIA

Childhood blindness is due to a group of diseases and conditions occurring in childhood or early adolescence (<16 years of age).^1^ Childhood blindness is a priority because of the number of years of blindness. It is estimated to be the second leading cause of years of blindness after cataract.^2^

Mission For Vision, in collaboration with Dr. Shroff's Charity Eye and ENT Hospital (SCEH), New Delhi has launched Mission Roshni in the year 2015. The purpose of this initiative is to ensure that all children aged 0–16 years in villages of Sardhana and Daurala blocks in Meerut district of Uttar Pradesh are screened and provided with necessary and adequate eye health services.

## The setting: Why Uttar Pradesh?

Meerut is a bustling town in the populous state of Uttar Pradesh (UP), where 16.5% of Indians live. With a literacy rate of 56%, life expectancy of 60 years and infant mortality of 75/1,000 children, UP is ranked 15th amongst the Indian states on the Human Development Index (HDI) as per the Planning Commission's 2008 estimates.^3^ The under-five mortality rate in UP was 78 deaths per 1,000 live births which is the highest in India.^4^ Almost eight million people in UP live below the poverty line, constituting over one-fifth of the total poor in the country. UP fairs badly in terms of basic health care, though there are wide inter-region and inter-district variations. In education, UP registered the highest proportion of children aged six to 14 years who were out-of-school in 2016. UP also has the lowest school attendance rates of children, at 50–60%, along with Bihar, Manipur, West Bengal and Madhya Pradesh.^5^

## The intervention: Mission Roshni

According to the 2011 census, Meerut district's population was 3,443,689.^6^,^7^ The intervention concentrated in two distinct administrative blocks in Meerut district – Sardhana and Daurala. The objectives of Mission Roshni were to:

- Provide comprehensive eye health services to about 40,000 children annually in the age group (0 – 16 years) with any eye condition. The children included those who are enrolled in schools and *madrasas* (a school for Islamic instruction) and also those who are school dropouts.
- Build the capacity of 200 teachers, 550 Accredited Social Health Activists (ASHA) and *anganwadi* centre staff to identify and refer children with various eye conditions.
- Raise awareness about childhood blindness and promote increased utilisation of eye care services for children through the involvement of the community.

In the two years since its inception, children in both government and private schools and *madrasas* were screened by trained optometrists for ocular morbidities including visual acuity. Apart from screening children in schools and *madrasas*, efforts were made to reach out to those who were out-of-school by visiting homes in these two administrative blocks with the help of ASHAs and *anganwadi* workers. In order to ensure comprehensive coverage of eye-care services in the region, Mission Roshni enhanced the capacities of non-medical personnel who regularly interacted with the children – teachers, ICDS functionaries, ASHAs and *anganwadi* workers and family members of these children, to identify children with eye health conditions.

## Programme strategy

### Cluster-based approach

The approach of providing primary eye care at the community level in rural and underserved urban areas is a promising strategy in creating awareness and reducing the burden of avoidable eye diseases.^8^ The entire project intervention area was clustered into different zones, within which training of teachers and stakeholders, screening of children and provisioning of services were undertaken. This cluster approach helped concentrate work in a systematic manner and optimised the resources available, thereby leading to greater programme efficiency and results.

### Integrated primary eye care service approach

The project team consisted of a well-trained paediatric counsellor and a dedicated full-time paediatric ophthalmologist along with others. The team also recruited local *anganwadi* and ASHA workers to engage with the community. This integrated approach to primary child eye health facilitated complete coverage of children enrolled in schools and *madrasas* as well as those who were out-of-school. Building the capacities of school teachers and community level volunteers like ASHAs, proved to be beneficial in tapping and channeling paediatric patients to avail primary eye care services in local communities. By doing so Mission Roshni envisages improved uptake of eye health services in the region in future.

**Table 1:** Achievements of Mission Roshni in two years.

Indicator	Outcome
Children aged 0–6 years screened	12,906
Children aged 6–16 years screened in schools and *madrasas*	59,826
Out-of-school children aged 6–16 years screened	16,701
Total number of children aged 0–16 years screened	89,433
Number of schools and *madrasas* where screening was completed	283
Number of teachers trained	662
Number of *anganwadi* workers (AWW) trained	460
Children identified with refractive errors	3,161
Percentage of children identified with refractive errors	3.5%
Children provided with corrective glasses for refractive errors	3,147
Children identified with low vision	10
Children provided with low vision devices	7
Children identified for surgical treatment	139
Surgical treatment – Retina	3 (2.2%)
Surgical treatment – Strabismus	103 (74.1%)
Surgical treatment – Cataract	22 (15.8%)
Surgical treatment – Ptosis	9 (6.5%)
Surgical treatment – Dacryocystorhinostomy (DCR)	1 (0.7%)
Surgical treatment – Secondary intra-ocular lens (IOL)	1 (0.7%)
Children provided with free surgical treatment	136
Number of family members counselled	4,090
Number of family members re-contacted for follow-up counselling	352
Number of community meetings held	270

### Counseling for Behavior change

Public health programmes can only deliver benefits if they are able to sustain activities over time. Refractive error is a leading cause of avoidable visual impairment globally, and India is not an exception. Children with refractive errors are prescribed appropriate spectacles which significantly improve their functionality and productivity. However, many studies point to compliance with spectacle use as an issue that is overlooked. One of Mission Roshni's core strategies was to have a dedicated full-time paediatric counsellor in place who would provide regular counselling to children and their immediate family members in order to ensure uptake of vision correction services and improve compliance with spectacle use.

## Way forward

Mission Roshni has achieved its desired results. The implementation approaches that were attempted have proven their merits while also showing different ways of working. These approaches can certainly be replicated in other geographic zones. While the deliverables may vary depending on the need of the area, the clustered approach with the help of different stakeholders in the community, coupled with quality service provision is surely the approach that would yield desired and sustained intervention impacts.

## Acknowledgements

The authors wish to thank the staff at our partner hospital, Dr. Shroff's Charity Eye and ENT Hospital, New Delhi for their support in planning, designing and implementing the Mission Roshni project in Meerut district in Uttar Pradesh.
